# Supplementary material for: Time-resolved multi-omics reveals diverse metabolic strategies of Salmonella during diet-induced inflammation
Source: mSphere. 2024 Sep 10;9(10):e00534-24. doi: 10.1128/msphere.00534-24 (PMC11520297; doi:10.1128/msphere.00534-24)
Supplement: Supplemental Figures — Figures S1 to S7. [file msphere.00534-24-s0005.pdf]

Supplemental Figures and Legends

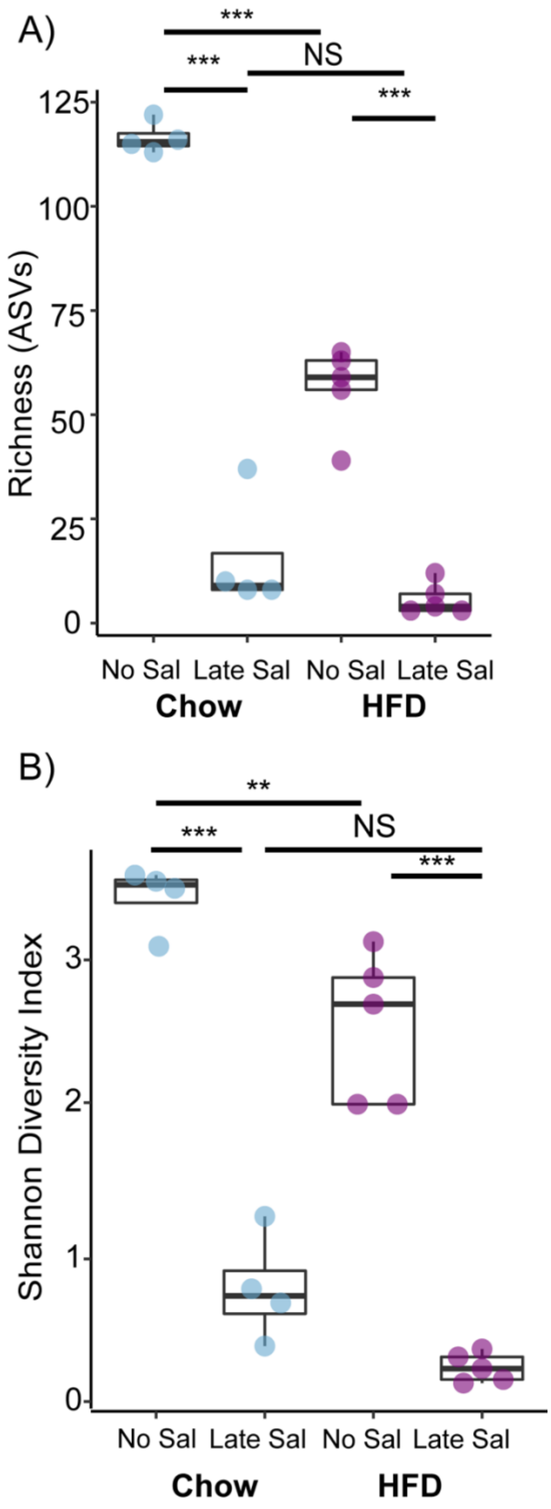

Figure S1: Microbial Diversity between Chow and HFD mice

A) Richness and B) Shannon's diversity metrics between chow (Chow) and high-fat diet (HFD) mice fecal samples prior to infection (day -1) and during late infection (day 11 or day 8, respectively). Asterisks indicate statistical significance where ** is a *p* value of <0.01 and *** is a *p* value of <0.001. NS indicates that there is no statistically significant difference.

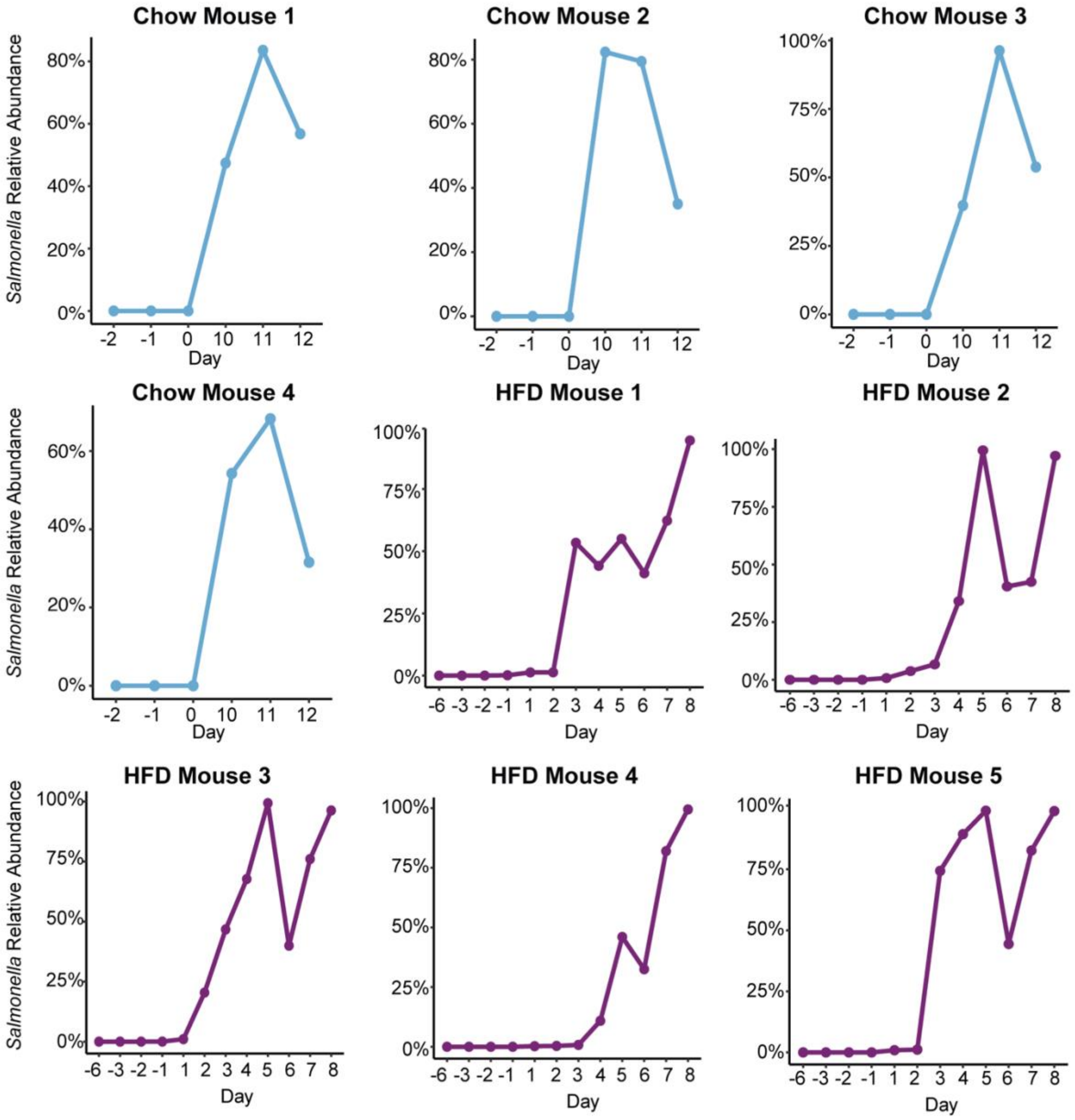

Figure S2: 16S *Salmonella* relative abundance per mouse in the Chow and High-fat Diet

The line plots show 16S rRNA gene relative abundance of *Salmonella* per mouse in the Chow and high-fat diet over time.

A)

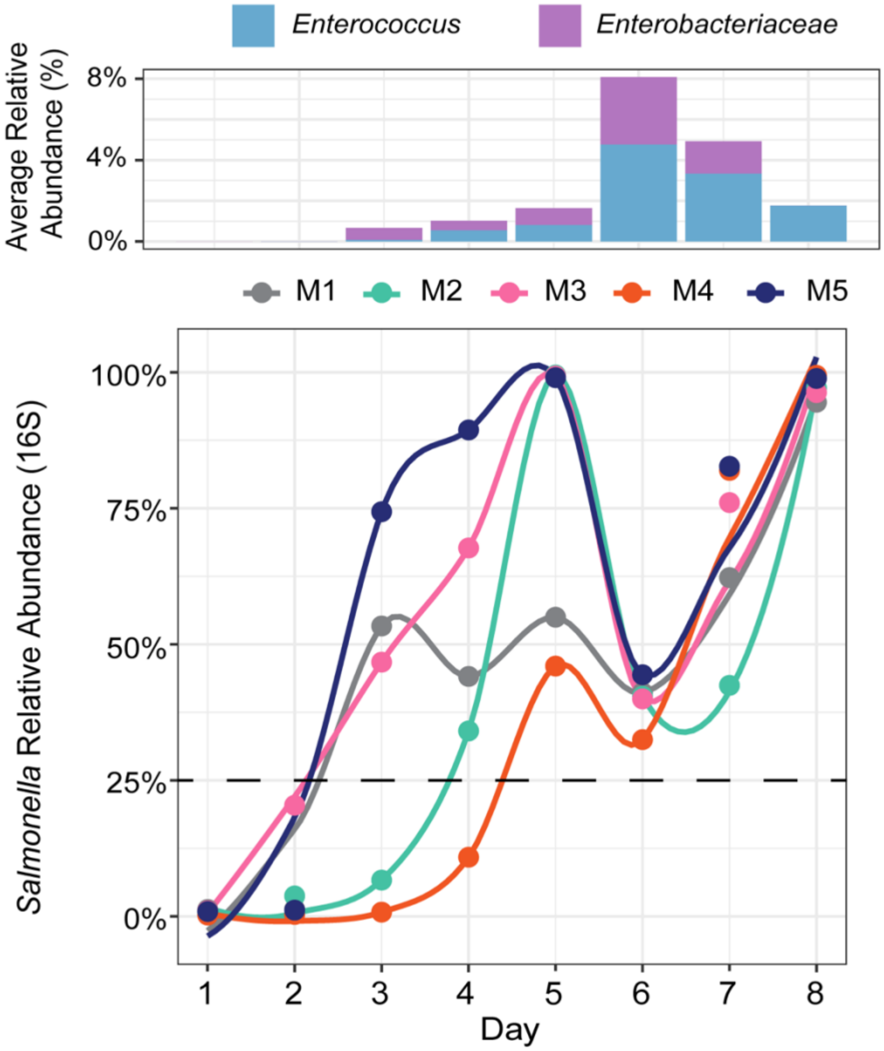

B)

Cecal CFU Counts per Mouse (Day 9)

Mouse	M1	M2	M3	M4	M5
Cecal CFU Counts (CFU/ml)	1.2×10^7	4.9×10^8	3.0×10^7	4.9×10^8	2.4×10^8

Figure S3: 16S *Salmonella* relative abundance and CFUs per mouse over time in the High-fat Diet

A) The line plot (bottom) shows 16S rRNA gene relative abundance of *Salmonella* per mouse over time, and the bar chart (top) shows the average relative abundance of one *Enterococcus* amplicon sequencing variant (ASV) and one Enterobacteriaceae ASV over time. B) Table shows cecal colony forming units (CFU/ml) counts per mouse post-sacrifice (day 9).

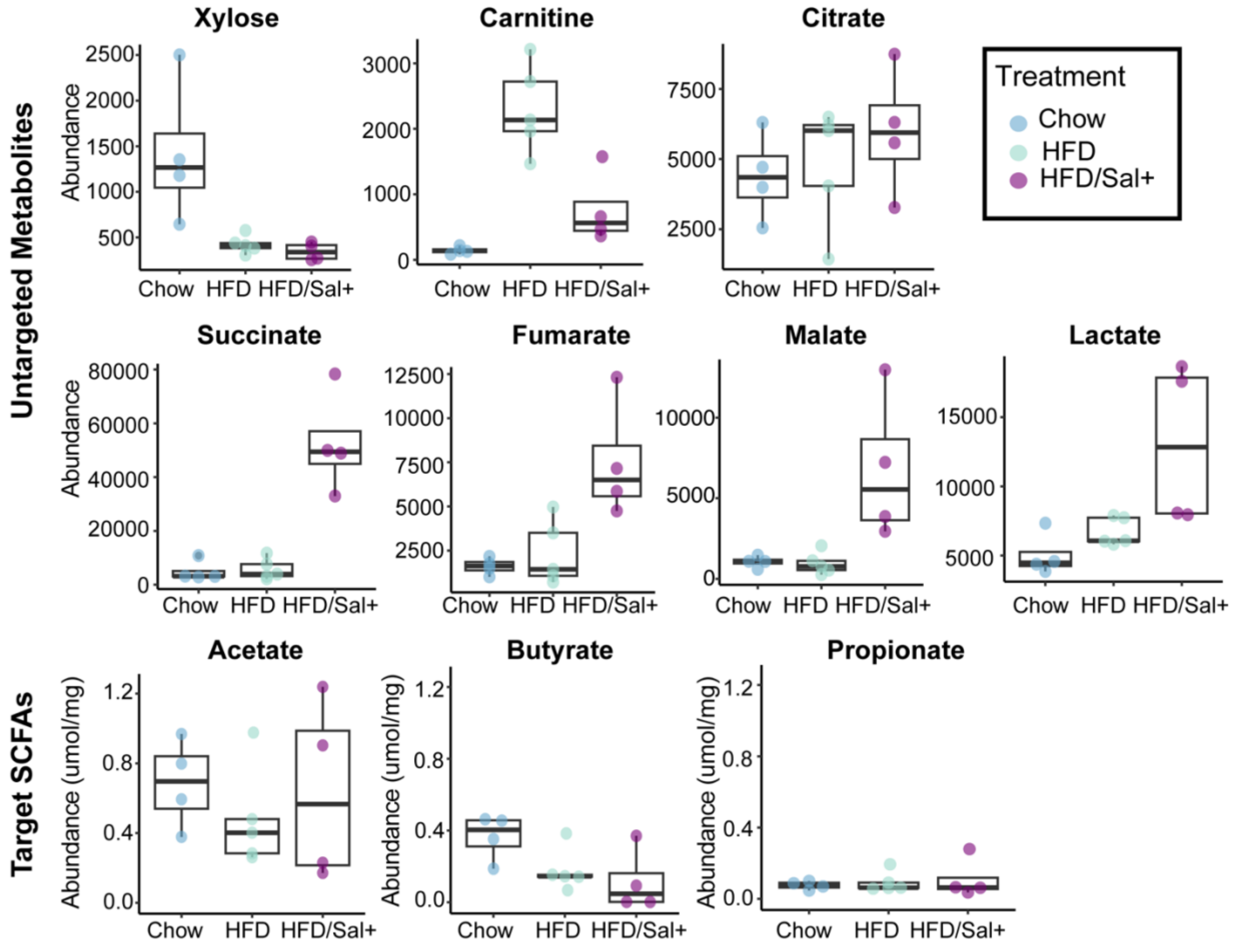

Figure S4: Metabolite abundance during late infection phase across treatments

Box plots depict the untargeted (xylose= C00181, carnitine= C00487, aspartate=C00049, citrate= C02226, succinate= C00042, fumarate= C00122, malate= C00497, lactate= C00256) and targeted (acetate, butyrate, propionate) median and Q1/Q3 -/+ 1.5 interquartile range of metabolite abundance in fecal samples of

uninfected chow (Chow, blue), uninfected high-fat diet (HFD, light green), and infected high-fat diet (HFD/Sal+, purple) mice on day 7. Asterisks indicate statistical significance where * is a p value of <0.05, ** is a p value of <0.01, and *** is a p value of <0.001.

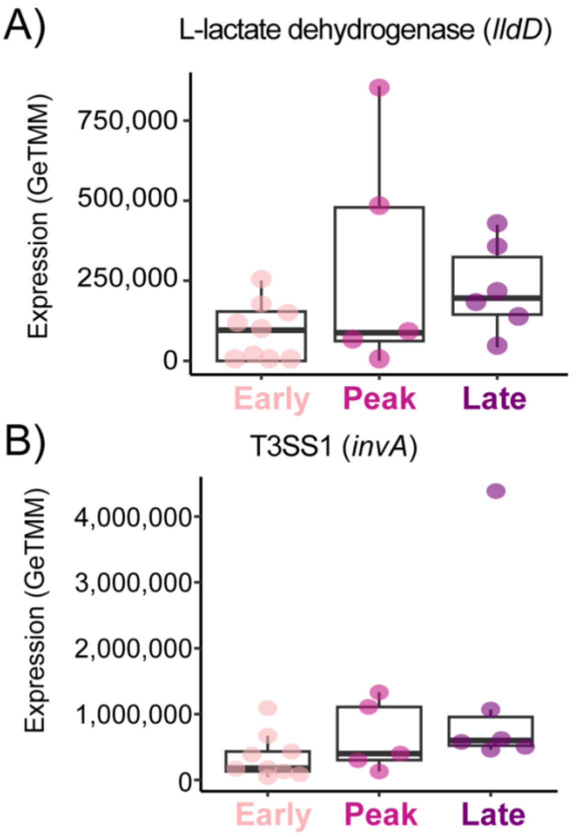

C)

Genes	Mean (GeTMM)	Median (GeTMM)
lldD Early	90,150	95,657
lldD Peak	297,150	87,595
lldD Late	225,588	196,156
invA Early	355,165	179,540
invA Peak	654,175	400,584
invA Late	225,588	196,156

Figure S5: Other active, non-differentially expressed genes

Box plots show the median and Q1/Q3 -/+ 1.5 interquartile range of normalized gene expression of A) L-lactate dehydrogenase (*lldD*), B) a type III secretion protein (*invA*) across infection phases and C) a table of the mean and median expression values for L-lactate dehydrogenase (*lldD*) and a type III secretion protein (*invA*).

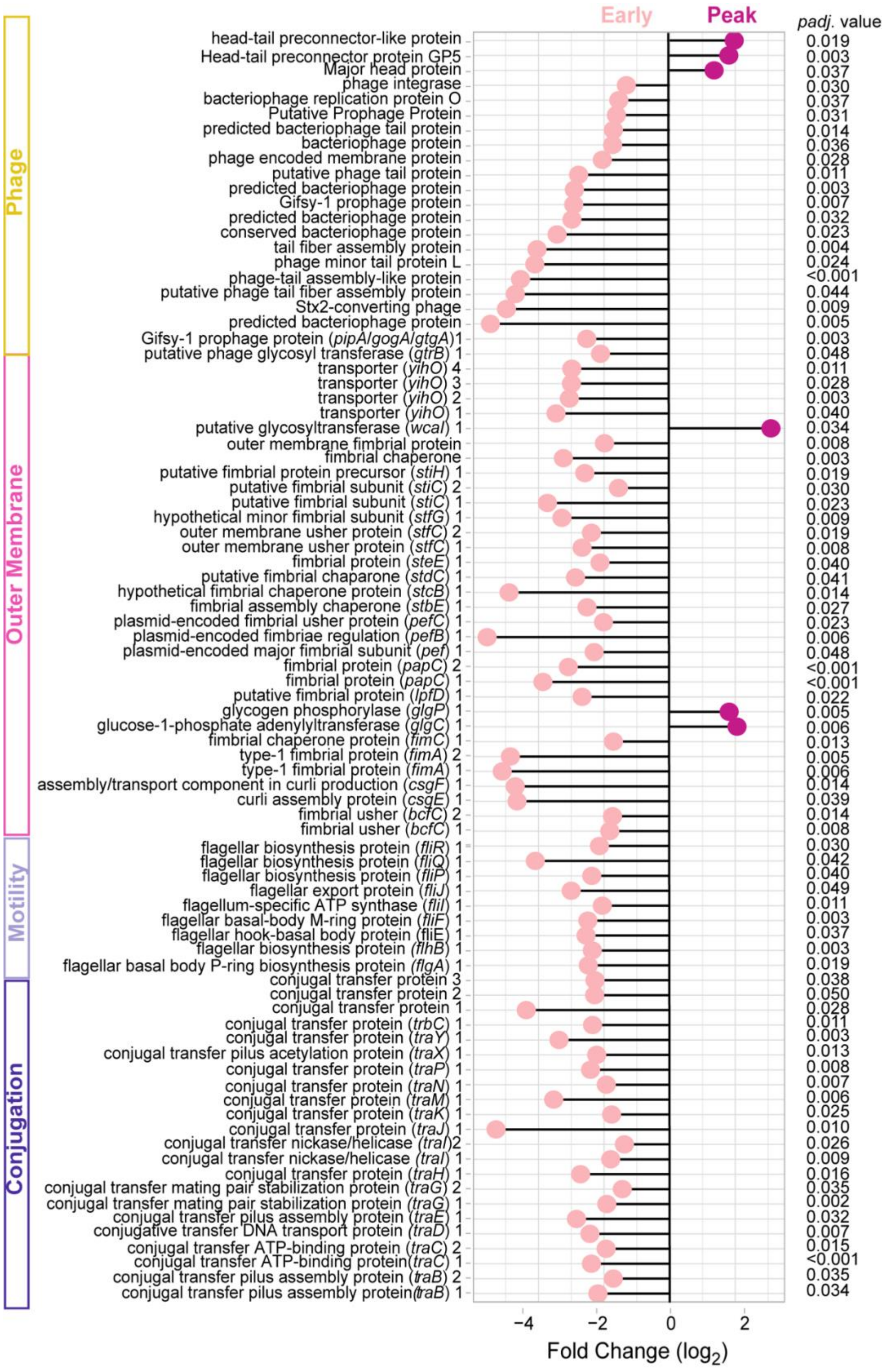
Early
Peak
padj. value
Phage
Outer Membrane
Motility
Conjugation
head-tail preconnector-like protein 0.019
Head-tail preconnector protein GP5 0.003
Major head protein 0.037
phage integrase 0.030
bacteriophage replication protein O 0.037
Putative Prophage Protein 0.031
predicted bacteriophage tail protein 0.014
bacteriophage protein 0.036
phage encoded membrane protein 0.028
putative phage tail protein 0.011
predicted bacteriophage protein 0.003
Gifsy-1 prophage protein 0.007
predicted bacteriophage protein 0.032
conserved bacteriophage protein 0.023
tail fiber assembly protein 0.004
phage minor tail protein L 0.024
phage-tail assembly-like protein <0.001
putative phage tail fiber assembly protein 0.044
Stx2-converting phage 0.009
predicted bacteriophage protein 0.005
Gifsy-1 prophage protein (pipA/gogA/gtgA)1 0.003
putative phage glycosyl transferase (gtrB) 1 0.048
transporter (yihO) 4 0.011
transporter (yihO) 3 0.028
transporter (yihO) 2 0.003
transporter (yihO) 1 0.040
putative glycosyltransferase (wcaI) 1 0.034
outer membrane fimbrial protein 0.008
fimbrial chaperone 0.003
putative fimbrial protein precursor (stiH) 1 0.019
putative fimbrial subunit (stiC) 2 0.030
putative fimbrial subunit (stiC) 1 0.023
hypothetical minor fimbrial subunit (stfG) 1 0.009
outer membrane usher protein (stfC) 2 0.019
outer membrane usher protein (stfC) 1 0.008
fimbrial protein (steE) 1 0.040
putative fimbrial chaparone (stdC) 1 0.041
hypothetical fimbrial chaperone protein (stcB) 1 0.014
fimbrial assembly chaperone (stbE) 1 0.027
plasmid-encoded fimbrial usher protein (pefC) 1 0.023
plasmid-encoded fimbriae regulation (pefB) 1 0.006
plasmid-encoded major fimbrial subunit (pef) 1 0.048
fimbrial protein (papC) 2 <0.001
fimbrial protein (papC) 1 <0.001
putative fimbrial protein (lpfD) 1 0.022
glycogen phosphorylase (glgP) 1 0.005
glucose-1-phosphate adenylyltransferase (glgC) 1 0.006
fimbrial chaperone protein (fimC) 1 0.013
type-1 fimbrial protein (fimA) 2 0.005
type-1 fimbrial protein (fimA) 1 0.006
assembly/transport component in curli production (csgF) 1 0.014
curli assembly protein (csgE) 1 0.039
fimbrial usher (bcfC) 2 0.014
fimbrial usher (bcfC) 1 0.008
flagellar biosynthesis protein (fliR) 1 0.030
flagellar biosynthesis protein (fliQ) 1 0.042
flagellar biosynthesis protein (fliP) 1 0.040
flagellar export protein (fliJ) 1 0.049
flagellum-specific ATP synthase (fliI) 1 0.011
flagellar basal-body M-ring protein (fliF) 1 0.003
flagellar hook-basal body protein (fliE) 1 0.037
flagellar biosynthesis protein (flhB) 1 0.003
flagellar basal body P-ring biosynthesis protein (flgA) 1 0.019
conjugal transfer protein 3 0.038
conjugal transfer protein 2 0.050
conjugal transfer protein 1 0.028
conjugal transfer protein (trbC) 1 0.011
conjugal transfer protein (traY) 1 0.003
conjugal transfer pilus acetylation protein (traX) 1 0.013
conjugal transfer protein (traP) 1 0.008
conjugal transfer protein (traN) 1 0.007
conjugal transfer protein (traM) 1 0.006
conjugal transfer protein (traK) 1 0.025
conjugal transfer protein (traJ) 1 0.010
conjugal transfer nickase/helicase (traI)2 0.026
conjugal transfer nickase/helicase (traI) 1 0.009
conjugal transfer protein (traH) 1 0.016
conjugal transfer mating pair stabilization protein (traG) 2 0.035
conjugal transfer mating pair stabilization protein (traG) 1 0.002
conjugal transfer pilus assembly protein (traE) 1 0.032
conjugative transfer DNA transport protein (traD) 1 0.007
conjugal transfer ATP-binding protein (traC) 2 0.015
conjugal transfer ATP-binding protein(traC) 1 <0.001
conjugal transfer pilus assembly protein (traB) 2 0.035
conjugal transfer pilus assembly protein(traB) 1 0.034
−4
−2
0
2
Fold Change (log2)

Figure S6: Differential expression of pathogenesis genes between early and peak phases

Lollipop plot of fold change (log_2) of differentially expressed (DESeq2, *padj.* >0.05) pathogenesis pathway genes between early (light pink) and peak (dark pink) infection phases. Genes are ordered by gene categories: conjugation (purple), motility (light purple), outer membrane (pink), and phage-like genes (yellow). Adjusted *p* values are listed in the *padj*. value column.

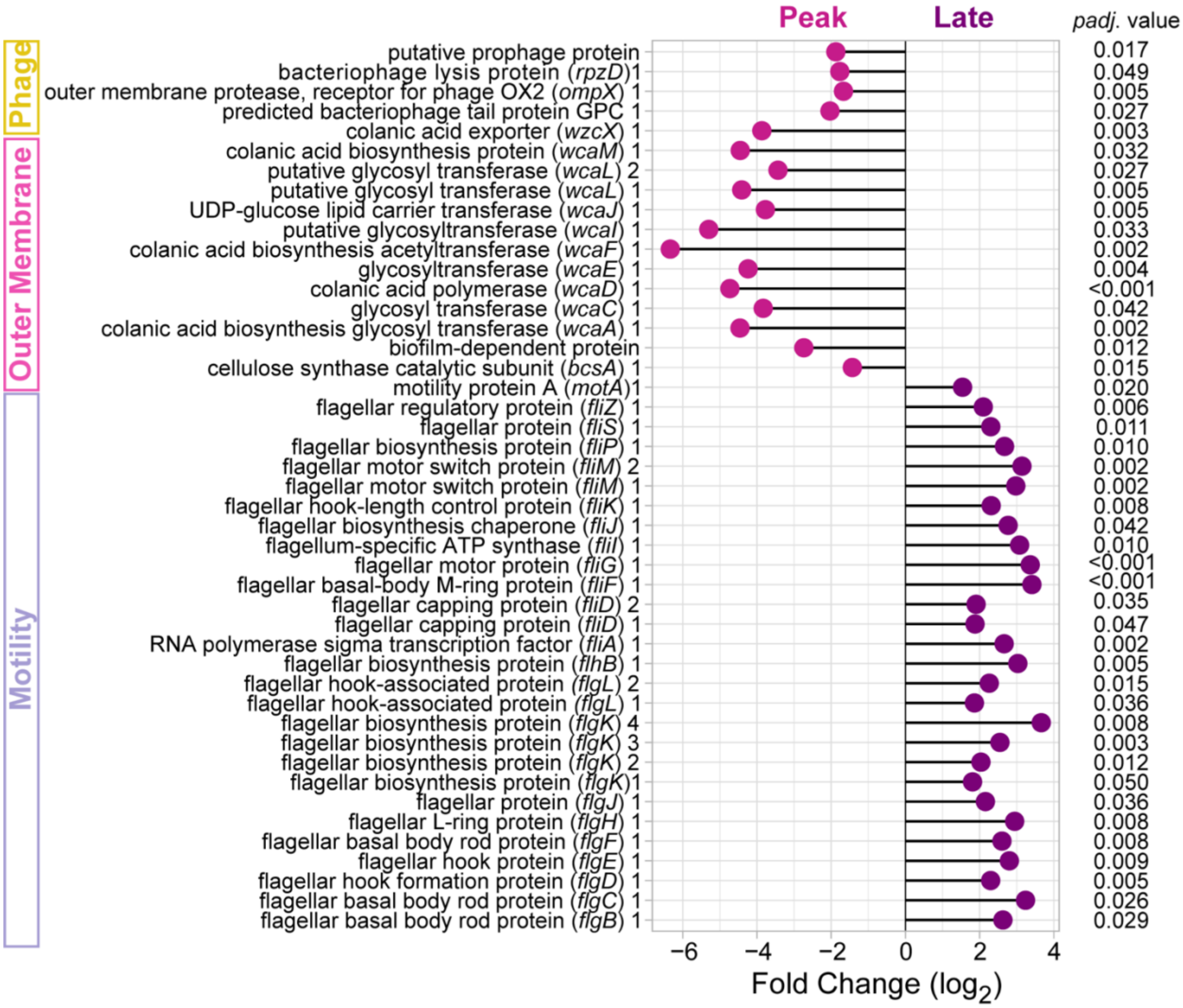

Figure S7: Differential expression of pathogenesis genes between peak and late phases

Lollipop plot of fold change (log_2) of differentially expressed (DESeq2, *padj.* >0.05) pathogenesis pathway genes between peak (dark pink) and late (purple) infection phases. Genes are ordered by gene categories: motility (light purple), outer membrane (pink), and phage-like genes (yellow). Adjusted *p* values are listed in the *padj*. value column.
